# Current Antithrombotic Prescribing Habits for Extended Secondary Prevention in Patients with Peripheral Artery Disease and Unprovoked Venous Thromboembolism: A Survey Among Specialists in Angiology and Vascular Surgery

**DOI:** 10.3390/jcm14145157

**Published:** 2025-07-21

**Authors:** Elena Butera, Frederikus Albertus Klok, Jamilla Goedegebuur, Angelo Porfidia, Behnood Bikdeli, Walter Ageno, Roberto Pola

**Affiliations:** 1 Percorso Trombosi, Dipartimento di Scienza dell’Invecchiamento, Ortopediche e Reumatologiche, Fondazione Policlinico Universitario A. Gemelli IRCCS, Università Cattolica del Sacro Cuore, 00168 Rome, Italyroberto.pola@unicatt.it (R.P.); 2 Department of Medicine—Thrombosis and Hemostasis, Leiden University Medical Center, 2333 Leiden, The Netherlands; 3 Division of Cardiovascular Medicine and Thrombosis Research Group, Brigham and Women’s Hospital, Harvard Medical School, Boston, MA 02115, USA; 4 Dipartimento di Medicina, Università di Padova, 35122 Padua, Italy

**Keywords:** venous thromboembolism, peripheral artery disease, anticoagulation, antiplatelet therapy, antithrombotic therapy

## Abstract

**Background:** Venous thromboembolism (VTE) is conventionally treated with anticoagulant therapy. In contrast, the core treatment for peripheral artery disease (PAD) is antiplatelet therapy. VTE and PAD share common risk factors and may occur in the same patient. Nonetheless, there is little evidence of the best antithrombotic regimen to use when the two conditions coexist, especially in terms of the extended prevention of major adverse cardiovascular events (MACE), major adverse limb events (MALE), and VTE recurrences. **Methods:** We conducted an online survey of members of the Italian Society of Angiology and Vascular Medicine (SIAPAV) to explore current prescribing habits for extended antithrombotic therapy in patients with PAD and unprovoked VTE. The survey included four clinical scenarios with variations in age, gender, bleeding risk, index VTE event, and severity of PAD. In all cases, patients had received anticoagulation for 6 months, and the key question was how to continue treatment beyond 6 months from the index VTE event. **Results:** A total of 174 clinicians participated to the survey. The most common choice was combining antiplatelet therapy with a direct oral anticoagulant (DOAC) at a low dose. Full-dose DOAC alone or antiplatelet therapy alone were less frequently chosen. Older age and high bleeding risk increased the preference for antiplatelet therapy alone. **Conclusions:** This survey highlights the marked variability in antithrombotic prescribing patterns among specialists in vascular medicine for patients with unprovoked VTE and concomitant PAD, reflecting the lack of evidence on optimal management in this specific setting. More research is needed to define the safest and most effective treatment strategies for patients with concurrent PAD and VTE.

## 1. Introduction

Venous thromboembolism (VTE), encompassing deep venous thrombosis (DVT) and pulmonary embolism (PE), is conventionally managed with anticoagulant therapy (ACT). When VTE is unprovoked, extended ACT is often prescribed beyond 6 months to prevent thrombotic recurrences [1]. Atherosclerotic cardiovascular diseases (ASCVD), such as peripheral artery disease (PAD), are instead usually treated with antiplatelet therapy (APT) [2]. In patients with PAD, APT is aimed at preventing major adverse cardiovascular events (MACE), such as cardiovascular death, myocardial infarction, coronary revascularization, and stroke [3]. APT in PAD also has the goal to prevent major adverse limb events (MALE), such as critical limb ischemia, peripheral revascularization, and limb amputation [4]. In recent years, there has been evidence that adding a “vascular” dose of rivaroxaban—a direct oral anticoagulant (DOAC) that inhibits coagulation Factor X—to standard APT provides further beneficial effects to patients with PAD in terms of both MACE and MALE [5]. Based on this, patients with PAD are currently treated with either APT alone or APT + a low dose of ACT (i.e., a “vascular” dose of rivaroxaban). This latter antithrombotic strategy is often referred to as dual pathway inhibition (DPI) [6].

VTE and ASCVD share common etiologic pathways and risk factors, and it is not uncommon that patients with PAD develop VTE, and vice versa [3]. We chose to focus on the case of concomitant PAD and unprovoked VTE because there is no high-quality data on the best antithrombotic management for these patients, especially for extended treatment [7,8,9]. The consequence of this is that patients are treated heterogeneously, with the risk that they are not sufficiently protected from arterial and venous thrombotic adverse events on one side and/or are exposed to increased bleeding risk on the other side. To explore current prescribing habits for extended antithrombotic therapy in patients with PAD and unprovoked VTE, we conducted an online survey among members of the Italian Society of Angiology and Vascular Medicine (SIAPAV).

## 2. Materials and Methods

We designed a survey with four different clinical scenarios representing patients with both PAD and unprovoked VTE. The cases were constructed to reflect common clinical situations with complex treatment decisions due to overlapping indications of APT and ACT. The cases varied based on the type of VTE event (DVT vs. intermediate–high risk PE) and the severity of PAD. In case 1, there was a 50-year-old man with intermittent claudication below 100 m (PAD stage IIb according to the Leriche–Fontaine classification) who had an unprovoked proximal DVT. In case 2, the patient was a 50-year-old woman with intermittent claudication (PAD stage IIb) and the VTE index event was an unprovoked intermediate–high risk PE. In case 3, there was a 50-year-old man with PAD that had undergone endovascular revascularization with stenting 1 year earlier and had an unprovoked proximal DVT. Case 4 differed from case 3 because the patient was a woman and the VTE index event was an unprovoked intermediate–high risk PE. In all scenarios, patients were receiving APT therapy prior to the index VTE event, which was discontinued at the time of VTE diagnosis, with concomitant prescription of DOAC therapy at full dose. The question for each case was which extended antithrombotic regimen would be most appropriate upon completion of 6 months of anticoagulation if the patient had no bleeding complications and normal kidney function. It was possible to choose among the following regimens: DOAC at full dose, APT alone, APT + eivaroxaban 2.5 mg *bis in die* (the DPI regimen), APT + low-dose DOAC, APT + full-dose DOAC, no treatment.

The rationale behind these case selections and possible answers was that an unprovoked VTE usually requires 6 months of ACT and then extended treatment with an anticoagulant at either full or reduced dose to prevent VTE recurrences [1]. The complication here is that these patients also have PAD, which is, at least theoretically, an indication of concomitant APT, or DPI. By distributing this survey, we wanted to explore whether specific clinical characteristics, such as VTE or PAD severity, might influence physicians’ choices for extended-phase therapy.

To further assess decision-making processes, in addition to the “default” scenarios described above, each clinical case had three possible variables: (i) opposite gender (a female rather than a male patient in cases 1 and 3; a male rather than a female patient in cases 2 and 4); (ii) the patient was >75-years-old rather than 50-years-old; (iii) the patient was at a high bleeding risk. Participants were asked to answer to all these variable scenarios. These modifiers were chosen because they reflect common real-life challenges that often influence the balance between thrombotic and bleeding risks in extended antithrombotic treatment decisions.

**Figure 1:**
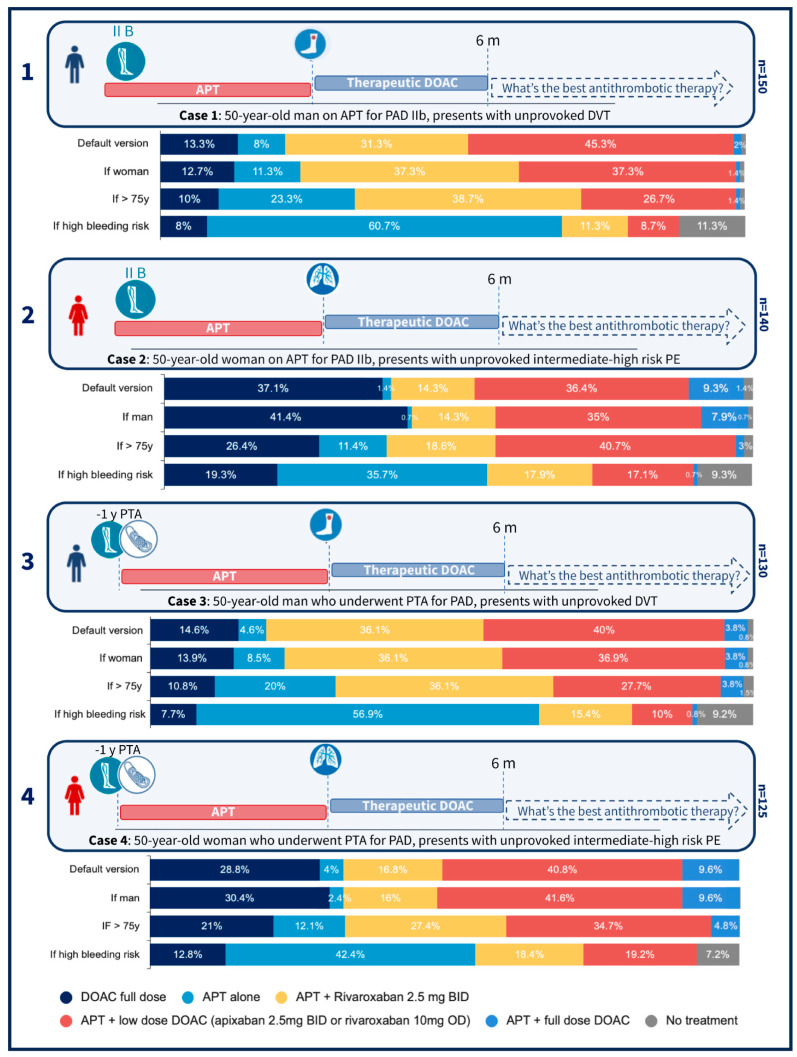
Illustration of the four clinical scenarios included in the survey, numbered from 1 to 4 on the left. Each scenario illustrates a patient (male or female) with peripheral artery disease (PAD), either stage IIb according to Leriche–Fontaine or with prior angioplasty one year earlier (-1y PTA), who experienced an unprovoked venous thromboembolism (either proximal DVT or intermediate–high-risk PE, indicated by a leg or lung icon, respectively). Antiplatelet therapy (APT) was discontinued at the time of the VTE event, and all patients received therapeutic DOAC for six months (6 m). Participants were then asked to choose the most appropriate antithrombotic strategy moving forward. The total number of respondents per scenario is shown on the right (*n* = x). Responses are reported as observed percentages and color-coded according to treatment choice, as indicated in the legend at the bottom. The “default version” refers to the core case summarized below each illustration; modified versions including opposite sex, advanced age, or high bleeding risk, were explored in subsequent questions.

The survey was distributed online among the members of the Italian Society of Angiology and Vascular Medicine (SIAPAV), which is the oldest and largest scientific society in the field of vascular medicine in Italy and comprises physicians with clinical expertise in both angiology and vascular surgery. The distribution occurred between December 2023 and February 2024. All survey responses were anonymous. We included both complete and partial responses in the analysis. As a result, the number of respondents varied slightly across questions, ranging from 174 for the initial professional background questions to 150 for the first clinical scenario and 125 for the last one. The number of respondents for each case is reported in Figure 1. To avoid the risk of duplicate entries, the survey platform restricted submissions to one per device. The complete survey is available in the online Supplementary Materials section.

## 3. Results

We collected answers from 174 physicians, who represent about 30% of the members of SIAPAV. The respondents were mainly specialists in internal medicine with a strong interest in vascular medicine (46%), angiologists (36%), and vascular surgeons (15%). More than 76% declared themselves to have more than 10 years of experience in their specialty field. All participants declared themselves to have clinical experience in the management of PAD patients. Response rates varied slightly across the four clinical scenarios, as shown in detail in Figure 1.

In the “default” version of the four clinical scenarios, respondents mainly opted for extended combination therapy with APT and a DOAC at low dose (apixaban 2.5 mg twice daily or rivaroxaban 10 mg once daily). This was the choice made by 45.3%, 36.4%, 40.0%, and 40.8% of respondents in cases 1, 2, 3, and 4, respectively. Although APT + DOAC at a low dose was the most frequent choice, it did not represent more than 50% of the answers in none of the proposed scenarios. There were indeed many physicians who opted for other therapeutic regimens, such as a DOAC at full dose without APT. This was the choice of 13.3%, 37.1%, 14.6%, and 28.8% of respondents in cases 1, 2, 3, and 4, respectively. There was also a considerable number of responders who chose APT + “vascular” dose rivaroxaban (2.5 mg twice daily) (the DPI regimen): 31.3%, 14.3%, 36.1%, and 16.8% % in cases 1, 2, 3, and 4, respectively.

Regarding the variables that were added to the “default” clinical scenarios, prescriptions were little influenced by the gender of the patient. Answers were instead substantially influenced by the age of the patient. Indeed, if the patient was older than 75 years, the prescription of APT alone increased from 8.0% to 23.3% in case 1, from 1.4% to 11.4% in case 2, from 4.6% to 20.0% in case 3, and from 4.0% to 12.1% in case 4. APT alone became the preferred option when the variable “high bleeding risk” was introduced: 60.7%, 35.7%, 56.9%, and 42.4% in cases 1, 2, 3, and 4, respectively. These findings reflect observed numerical trends rather than the results of formal statistical testing, as no inferential analyses were performed. Detailed percentages are shown in Figure 1.

## 4. Discussion

This survey shows that there is considerable variation in the preferred extended antithrombotic regimen to be used in patients with PAD and unprovoked VTE. The combination of APT with low-dose DOAC was the preferred therapy by many physicians in most cases. However, there were physicians who preferred to continue with DOAC at full dose alone (without APT), others with APT alone (without ACT), others with APT + “vascular” dose rivaroxaban (DPI), and others with APT + DOAC at full dose. Older age and high bleeding risk were associated with a substantial increment in the prescription of APT alone, but there were still physicians who preferred other options in these situations as well.

It is important to note that none of the treatment regimens outlined above are based on high-quality clinical or scientific evidence; therefore, there is no demonstration that one treatment should be preferred to another in all cases. Indeed, the net clinical benefit of APT alone, ACT alone, or APT + ACT at any dose (either full, reduced, or at “vascular dose”) in the set of patients considered in this survey is unknown. What is known is that patients with stable coronary artery disease (CAD) and atrial fibrillation (AF) should be treated in the long term with full-dose ACT alone (either warfarin or a DOAC), without a concomitant antiplatelet agent, to minimize bleeding risk [10,11,12]. However, the combination of PAD and unprovoked VTE addressed in this survey is different from the combination of CAD and AF due to fundamental differences in pathophysiology and therapeutic objectives. First, the mechanism and site of thrombus formation in AF versus PAD/VTE differ substantially, therefore warranting different treatment strategies. In AF, thrombi originate from abnormal flow in the left atrial appendage, requiring ATC treatment [13]. In PAD, arterial thrombosis mainly arises from endothelial activation, plaque rupture, and platelet aggregation—justifying antiplatelet agents or DPI [14]. Conversely, VTE arises in venous systems due to coagulation cascade activation, thus responding primarily to ATC [15]. Second, the duration of treatment differs, since AF mandates life-long anticoagulation based on stroke risk scores such as CHA_2_DS_2_-VASc [16], while unprovoked VTE has a definite full-dose ATC initial treatment phase, often followed by extended treatment with reduced doses of DOACs [17]. Additionally, patients with PAD have peculiar characteristics: they are at high risk not only of MACE but also of MALE, different from patients with CAD without PAD [4]. Patients with PAD also have a poorer prognosis compared to other individuals with ASCVD, including CAD [18]. For these reasons, it cannot be taken for granted that extended ACT alone is enough to prevent adverse arterial events in PAD patients. Support for this concept comes from the DPI trials, such as the COMPASS and the VOYAGER studies [5,19,20], as well as real-world data on PAD patients treated with DPI, such as the XATOA Registry [21]. Based on these data, extended DPI rather than ACT might be particularly attractive in PAD patients at very high risk for cardiovascular events, such as those with previous peripheral revascularization, previous critical limb ischemia, prior carotid artery revascularization, or carotid stenosis ≥ 50%. In conclusion, extrapolating CAD-AF evidence to the PAD-VTE overlap is inappropriate, and the heterogeneity we observed in prescribing habits likely reflects the absence of dedicated PAD-VTE guidance.

This study has a number of limitations. The number of physicians who completed the survey is relatively low. Since the survey was anonymous, we did not collect information on non-responders and are therefore unable to compare the characteristics of respondents and non-respondents. In addition, the survey was distributed only to Italian physicians; thus, the generalizability of our findings is limited. Furthermore, the results are based on self-reported prescribing attitudes, which may not fully reflect real-world clinical practice. Future research should consider extending the survey to international cohorts to allow for broader comparisons and to better understand global prescribing patterns. The four scenarios that we proposed are a simplification of clinical practice and do not include all the PAD and VTE combinations that may possibly occur. The same is true for the variables that we added to the “default” scenarios. Many other variables might be proposed. Regarding the “high bleeding risk” variable, we did not specify a precise situation or provide standardized criteria (e.g., validated bleeding risk score); instead, we left the interpretation of this concept to the individual respondent. While this approach allowed us to reflect real-life variability in clinical judgment, it also introduces subjectivity and potential heterogeneity in how the scenario was perceived. This may limit the comparability of responses related to this variable. Thus, caution should be used when interpreting these results. For future studies, a more structured definition of bleeding risk would improve internal consistency and enhance interpretability. Finally, our survey focused on extended treatment beyond 6 months from an index VTE event. However, it should be noted that clear indications do not exist for treatment within the first 6 months of an index VTE event in PAD patients either. This issue is the object of dedicated investigations, such as the BAT-VTE trial, which is currently enrolling patients to assess if stopping APT is safer than combining this treatment with full-dose ACT for an acute VTE in patients receiving aspirin or another APT for the secondary prevention of cardiovascular diseases [22].

In conclusion, our findings demonstrate that vascular physicians have very heterogeneous prescribing habits when dealing with patients with PAD who experience unprovoked VTE events. This reflects the lack of evidence on this topic in the medical literature. There is an urgent need for more targeted research to help clinicians optimize antithrombotic strategies and improve the outcomes of concomitant PAD and unprovoked VTE.

## Data Availability

The raw data supporting the conclusions of this article will be made available by the authors on request.

## Supplementary Materials

The following supporting information can be downloaded at https://www.mdpi.com/article/10.3390/jcm14145157/s1, Full text survey.

## Abbreviations

The following abbreviations are used in this manuscript:

VTE	Venous Thromboembolism
DVT	Deep Vein Thrombosis
PE	Pulmonary Embolism
ACT	Anticoagulant Therapy
ASCVD	Atherosclerotic Cardiovascular Diseases
APT	Antiplatelet Therapy
DOAC	Direct Oral Anticoagulant
SIAPAV	Italian Society for Angiology and Vascular Medicine
