# Supplementary material for: Current Antithrombotic Prescribing Habits for Extended Secondary Prevention in Patients with Peripheral Artery Disease and Unprovoked Venous Thromboembolism: A Survey Among Specialists in Angiology and Vascular Surgery
Source: J Clin Med. 2025 Jul 21;14(14):5157. doi: 10.3390/jcm14145157 (PMC12295201; doi:10.3390/jcm14145157)
Supplement: Supplementary file 1 [file jcm-14-05157-s001.zip › jcm-3687552-supplementary.pdf]

SURVEY

Unprovoked venous thromboembolism in subjects with an established atherosclerotic cardiovascular disease: what is the best antithrombotic strategy after 6 months from the thromboembolic event? The case of peripheral artery disease

Case 1
A 50-year-old man suffers from **symptomatic lower limb peripheral artery disease (PAD) with intermittent claudication** (Fontaine stages IIb). Six months ago, he was diagnosed with idiopathic **deep vein thrombosis (DVT) of the right popliteal vein**. His kidney function is normal. Upon DVT diagnosis, therapy with antiplatelet therapy (APT), which the patient was taking for the treatment of PAD, was discontinued, and anticoagulant therapy with a direct oral anticoagulant (DOAC) was started. The patient has so far received six months of uninterrupted anticoagulant treatment.

1A. What antithrombotic treatment would you recommend for this patient from now on?
a) Continue full dose DOAC monotherapy
b) Stop DOAC and restart APT
c) APT + vascular dose rivaroxaban (2.5mg BID)
d) APT + reduced dose DOAC (apixaban 2.5mg BID or rivaroxaban 10mg OD)
e) APT + full dose DOAC
f) No antithrombotic treatment

1B. If the patient featured in clinical case 1 was a woman, what antithrombotic treatment would you recommend from now on?
a) Continue full dose DOAC monotherapy
b) Stop DOAC and restart APT
c) APT + vascular dose rivaroxaban (2.5mg BID)
d) APT + reduced dose DOAC (apixaban 2.5mg BID or rivaroxaban 10mg OD)
e) APT + full dose DOAC
f) No antithrombotic treatment

1C. If the patient featured in clinical case 1 was older than 75 years, what antithrombotic treatment would you recommend from now on?
a) Continue full dose DOAC monotherapy
b) Stop DOAC and restart APT
c) APT + vascular dose rivaroxaban (2.5mg BID)
d) APT + reduced dose DOAC (apixaban 2.5mg BID or rivaroxaban 10mg OD)
e) APT + full dose DOAC
f) No antithrombotic treatment

1D. If the patient featured in clinical case 1 had a high risk of bleeding, what antithrombotic treatment would you recommend from now on?
a) Continue full dose DOAC monotherapy
b) Stop DOAC and restart APT
c) APT + vascular dose rivaroxaban (2.5mg BID)
d) APT + reduced dose DOAC (apixaban 2.5mg BID or rivaroxaban 10mg OD)
e) APT + full dose DOAC
f) No antithrombotic treatment

Case 2
A 50-year-old woman suffers from **symptomatic lower limb peripheral artery disease (PAD) with intermittent claudication** (Fontaine stages IIb). Six months ago, she was diagnosed with **idiopathic intermediate-high risk pulmonary embolism (PE)**. Her kidney function is normal. Upon PE diagnosis, therapy with antiplatelet therapy (APT), which the patient was taking for the treatment of PAD, was discontinued, and anticoagulant therapy was started. The patient in now on treatment with a DOAC and has so far received anticoagulant treatment for a total of six months.

2A. What antithrombotic treatment would you recommend for this patient from now on?

a) Continue full dose DOAC monotherapy
b) Stop DOAC and restart APT
c) APT + vascular dose rivaroxaban (2.5mg BID)
d) APT + reduced dose DOAC (apixaban 2.5mg BID or rivaroxaban 10mg OD)
e) APT + full dose DOAC
f) No antithrombotic treatment

2B. If the patient featured in clinical case 2 was a man, what antithrombotic treatment would you recommend from now on?

a) Continue full dose DOAC monotherapy
b) Stop DOAC and restart APT
c) APT + vascular dose rivaroxaban (2.5mg BID)
d) APT + reduced dose DOAC (apixaban 2.5mg BID or rivaroxaban 10mg OD)
e) APT + full dose DOAC
f) No antithrombotic treatment

2C. If the patient featured in clinical case 2 was older than 75 years, what antithrombotic treatment would you recommend from now on?

a) Continue full dose DOAC monotherapy
b) Stop DOAC and restart APT
c) APT + vascular dose rivaroxaban (2.5mg BID)
d) APT + reduced dose DOAC (apixaban 2.5mg BID or rivaroxaban 10mg OD)
e) APT + full dose DOAC
f) No antithrombotic treatment

2D. If the patient featured in clinical case 2 had a high risk of bleeding what antithrombotic treatment would you recommend from now on?

a) Continue full dose DOAC monotherapy
b) Stop DOAC and restart APT
c) APT + vascular dose rivaroxaban (2.5mg BID)
d) APT + reduced dose DOAC (apixaban 2.5mg BID or rivaroxaban 10mg OD)
e) APT + full dose DOAC
f) No antithrombotic treatment

Case 3
A 50-year-old man suffers from **lower limb peripheral artery disease (PAD)** for which he underwent a **percutaneous transluminal angioplasty (PTA) with placement of a 7 cm stent in the left femoral superficial artery 1 year ago**. Six months ago, he was diagnosed with **idiopathic deep vein thrombosis (DVT) of the right popliteal vein**. His kidney function is normal. Upon DVT diagnosis, therapy with antiplatelet therapy (APT), which the patient was taking for the treatment of PAD, was discontinued, and anticoagulant therapy with a DOAC was started. The patient has so far received six months of anticoagulant treatment.

3A. What antithrombotic treatment would you recommend for this patient from now on?
a) Continue full dose DOAC monotherapy
b) Stop DOAC and restart APT
c) APT + vascular dose rivaroxaban (2.5mg BID)
d) APT + reduced dose DOAC (apixaban 2.5mg BID or rivaroxaban 10mg OD)
e) APT + full dose DOAC
f) No antithrombotic treatment

3B. If the patient featured in clinical case 3 was a woman, what antithrombotic treatment would you recommend from now on?
a) Continue full dose DOAC monotherapy
b) Stop DOAC and restart APT
c) APT + vascular dose rivaroxaban (2.5mg BID)
d) APT + reduced dose DOAC (apixaban 2.5mg BID or rivaroxaban 10mg OD)
e) APT + full dose DOAC
f) No antithrombotic treatment

3C. If the patient featured in clinical case 3 was older than 75 years, what antithrombotic treatment would you recommend from now on?
a) Continue full dose DOAC monotherapy
b) Stop DOAC and restart APT
c) APT + vascular dose rivaroxaban (2.5mg BID)
d) APT + reduced dose DOAC (apixaban 2.5mg BID or rivaroxaban 10mg OD)
e) APT + full dose DOAC
f) No antithrombotic treatment

3D. If the patient featured in clinical case 3 had a high risk of bleeding, what antithrombotic treatment would you recommend from now on?
a) Continue full dose DOAC monotherapy
b) Stop DOAC and restart APT
c) APT + vascular dose rivaroxaban (2.5mg BID)
d) APT + reduced dose DOAC (apixaban 2.5mg BID or rivaroxaban 10mg OD)
e) APT + full dose DOAC
f) No antithrombotic treatment

Case 4
A 50-year-old woman suffers from **lower limb peripheral artery disease (PAD)** for which she underwent a **percutaneous transluminal angioplasty (PTA) with placement of a 7 cm stent in the left femoral superficial artery 1 year ago**. Six months ago, she was diagnosed with **idiopathic intermediate-high risk pulmonary embolism (PE)**. Her kidney function is normal. Upon PE diagnosis, therapy with antiplatelet therapy (APT), which the patient was taking for the treatment of PAD, was discontinued, and anticoagulant therapy was started. The patient in now on treatment with a DOAC and has so far received anticoagulant treatment for a total of six months.

4A. What antithrombotic treatment would you recommend for this patient from now on?
a) Continue full dose DOAC monotherapy
b) Stop DOAC and restart APT
c) APT + vascular dose rivaroxaban (2.5mg BID)
d) APT + reduced dose DOAC (apixaban 2.5mg BID or rivaroxaban 10mg OD)
e) APT + full dose DOAC
f) No antithrombotic treatment

4B. If the patient featured in clinical case 3 was a man, what antithrombotic treatment would you recommend from now on?
a) Continue full dose DOAC monotherapy
b) Stop DOAC and restart APT
c) APT + vascular dose rivaroxaban (2.5mg BID)
d) APT + reduced dose DOAC (apixaban 2.5mg BID or rivaroxaban 10mg OD)
e) APT + full dose DOAC
f) No antithrombotic treatment

4C. If the patient featured in clinical case 4 was older than 75 years, what antithrombotic treatment would you recommend from now on?
a) Continue full dose DOAC monotherapy
b) Stop DOAC and restart APT
c) APT + vascular dose rivaroxaban (2.5mg BID)
d) APT + reduced dose DOAC (apixaban 2.5mg BID or rivaroxaban 10mg OD)
e) APT + full dose DOAC
f) No antithrombotic treatment

4D. If the patient featured in clinical case 4 had a high risk of bleeding, what antithrombotic treatment would you recommend from now on?
a) Continue full dose DOAC monotherapy
b) Stop DOAC and restart APT
c) APT + vascular dose rivaroxaban (2.5mg BID)
d) APT + reduced dose DOAC (apixaban 2.5mg BID or rivaroxaban 10mg OD)
e) APT + full dose DOAC
f) No antithrombotic treatment
